# (Non-)Sense of Milk Testing in Small Ruminant Lentivirus Control Programs in Goats. Comparative Analysis of Antibody Detection and Molecular Diagnosis in Blood and Milk

**DOI:** 10.3390/v12010003

**Published:** 2019-12-18

**Authors:** Nadjah Radia Adjadj, Jo Vicca, Rodolphe Michiels, Nick De Regge

**Affiliations:** 1 Unit of Enzootic, Vector-Borne and Bee Diseases, Sciensano, Groeselenberg 99, 1180 Brussels, Belgium; rodolphe.michiels@sciensano.be (R.M.); nick.deregge@sciensano.be (N.D.R.); 2 Odisee vzw, University College KULeuven, Campus Sint-Niklaas, Hospitaalstraat 23, 9100 Sint-Niklaas, Belgium; jo.vicca@odisee.be

**Keywords:** SRLV, milk, diagnosis, ELISA, qPCR, control program

## Abstract

Small ruminant lentivirus (SRLV) control programs are mainly based on diagnostic tests performed on blood samples collected from sheep and goats. Since blood sampling is costly and stressful for the animals, we evaluated whether milk could be used as an inexpensive and easily collectable matrix for SRLV detection. We therefore compared SRLV detection via two commercial enzyme-linked immunosorbent assays (ELISAs) and quantitative polymerase chain reaction (qPCR) in blood and corresponding milk samples from 321 goats originating from eight different SRLV-infected farms in Flanders (Belgium). The IDscreen^®^ ELISA had a better relative sensitivity (97% vs 93%) and specificity (100% and 97%) than the Elitest^®^ ELISA for SRLV-specific antibody detection in milk compared to serum. The higher sensitivity correlates with a 10-fold higher analytical sensitivity of the IDscreen^®^ test. In contrast to the overall good ELISA results, qPCR on milk cell pellets lacked sensitivity (81%) and specificity (88%), compared to molecular detection in blood leucocyte pellets. Our results show that serology is more suitable than qPCR for SRLV diagnosis, and that milk may represent an interesting matrix for a preliminary evaluation of a herd’s infection status. Serum remains however the sample of choice for control programs where it is important to identify positive animals with the highest sensitivity.

## 1. Introduction

Small ruminant lentiviruses (SRLVs) are retroviruses belonging to the *Lentivirus* genus. This group comprises the maedi-visna virus (*Visna virus*, MVV) and *Caprine arthritis-encephalitis virus* (CAEV). MVV and CAEV were first isolated from sheep and goats, respectively, but since it has been shown that both viruses may cross the species barrier, they were grouped together as SRLVs [1,2]. SRLVs are mainly transmitted via the ingestion of infected colostrum and milk by the offspring. Nevertheless, SRLVs were also detected in lung fluid, nasal secretions and saliva [2,3], and transmission may thus also occur via close contact and inhalation of infected secretions [4].

Monocytes and macrophages are the main target cells for SRLVs upon infection. After entry in the target cell, the viral RNA is reversely transcribed, and the resulting proviral DNA is integrated into the host’s genome, leading to a lifelong infection [5]. The infection of monocytes remains latent until their differentiation into macrophages occurs. This differentiation enhances the expression of different transcription factors, which expression triggers the transcription of proviral DNA and results in the production of new virus particles [6]. SRLV infection results in the activation of both the innate and adaptive immune system.

Viral antigens are presented to the adaptive immune system which responds among others by producing B-cell derived SRLV-specific antibodies. The development of the antibody response is however slow, and its efficiency is hindered by the high antigenic heterogeneity and immune evasion strategies of the virus. Likewise, this heterogeneity makes it challenging to design effective vaccines and diagnostic tools [2,5,7,8].

SRLVs cause a progressive infection leading to lesions in the lungs, mammary glands, central nervous system (CNS) and joints [6,9]. Besides the impact on animal health, SRLVs represent a serious economic threat to the small ruminant industry, since they result in decreased milk production, reduced lamb weight and restrictions to animal trade [6,10,11]. Some studies reported even a decline in fertility and number of lambs per birth in seropositive animals [6,12].

In the absence of vaccines, control programs are the sole approach to avoid the spread of SRLV infection. These programs rely mainly on strategies that prevent the introduction and/or the transmission of the virus [13]. Early diagnosis and good diagnostic tools are of crucial importance for the efficient prevention and control of SRLV infection. Enzyme-linked immunosorbent assay (ELISA) and agar gel immunodiffusion (AGID) are the most commonly used tests for the serological detection of SRLVs [13,14]. ELISAs are cost-effective, easily implemented and mostly show a good performance. AGID tests show a high specificity, but are mostly less sensitive compared to ELISA, and are not applied for a variety of diagnostic matrices [6,14,15]. This makes it a fact that AGID is usually used to confirm ELISA results [2]. All serological tests suffer from imperfect sensitivity that may be due to the heterogeneity of circulating strains, delayed seroconversion and fluctuating antibody responses [2,6,16]. Besides the serological tests, molecular assays were developed to enable the detection of viral nucleic acids. PCRs are the most frequently used methods and are a valuable tool for the detection of infected animals prior to antibody response. The low viral load in latently infected carriers and the high viral genetic heterogeneity however decrease PCR sensitivity [1,6,17]. Since no gold standard diagnostic method for SRLV detection exists, the combination of different tests is often necessary to detect the correct infection status of small ruminants [3,18].

Serological SRLV diagnosis is routinely carried out on serum, and leucocytes isolated from blood are mostly used for PCR-based diagnosis. Blood sampling however requires an invasive intervention by a veterinarian, which significantly increases the sampling costs. This raised the question of using milk as an alternative matrix, since it can easily and inexpensively be collected by the farmers [19,20,21], and is supposed to be able to provide similar information as blood about SRLV infection. Antibodies are namely transferred from blood into the mammary gland where they end up in colostrum or milk [22,23] and can also be locally produced in limited amounts by B cells in the mammary gland [22]. Furthermore, monocytes carrying proviral DNA can migrate from blood into the mammary gland, where they differentiate into macrophages and enter the mammary gland secretions [24,25,26]. Previous studies have investigated the usefulness of milk in the detection of SRLVs [19,20,21,27,28,29,30,31] and most reported a good agreement between antibody detection results in milk and blood [19,21,29,30]. Brinkhof et al. [21] have even indicated that milk is a suitable replacement for serum. The number of samples and farms tested remains however limited, and only little work was devoted to study molecular SRLV detection in milk via Real Time PCR (qPCR).

SRLVs are also present in Belgium, and a between-herd seroprevalence of 17% in sheep and 13% in goats farms was recently reported [32]. To provide farmers with the opportunity to participate at expositions and to export to third countries, the government has installed a voluntary program to obtain an SRLV-free certificate. The diagnostic decision tree used in this program was recently simplified based on the results of a comparative study performed at our lab [15], and now combines a first screening with the Elitest^®^ ELISA and confirmatory testing with the IDscreen^®^ ELISA and AGID tests. In the present study, we wanted to evaluate if the SRLV control program in Belgium could be further simplified by using milk instead of blood samples. We therefore performed a comparative analysis of SRLV antibody detection and molecular diagnosis via qPCR in both matrices on an extensive sample set.

## 2. Materials and Methods

### 2.1. Samples Origin

Blood and milk samples that were collected at the same time from 321 goats were used in this study. These represent all collected samples from 8 out of 12 organic goat farms in Flanders (Belgium) that had been shown to contain SRLV-positive animals during a study to determine the seroprevalence of SRLV, *Paratuberculosis* and *Cornebacterium pseudotuberculosis* (CL) in the Flemish organic goat sector (manuscript in preparation). In order to allow an estimate of the true seroprevalence of these diseases with 95% confidence and a precision of 5% based on an estimated prevalence of 50%, 620 samples were collected in total. The number of samples collected per farm was stratified based on the number of animals held per farm. An overview of the number of samples collected on each of the eight farms can be found in Table S1. The goat farmers participating in this study gave permission to conduct the study on their premises. No specific ethical dossier had to be filed for this study since the collection of blood and milk from goats at the farm by a veterinarian is considered as a routine veterinary practice and needs no specific approval from an ethical committee under current European and Belgian legislation (Directive 2010/63/EU of the European parliament and of the council of 22 September 2010 on the protection of animals used for scientific purposes; Belgian Royal Decree of May 2013 relating to the accommodation and care of experimental animals (C 2013/24221, chap I. §4)). 

### 2.2. Processing of Blood Samples

Blood collected in 10 mL serum tubes was centrifuged at 1500 rpm for 10 min. The obtained serum was aliquoted and stored at −20 °C until testing.

Blood collected in ethylenediaminetetraacetic acid (EDTA) tubes was used to produce leucocyte pellets. Eight ml of hemolysis buffer (16.6 g NH_4_Cl, 2.0 g NaHCO_3_, 0.185 g diNa EDTA per L H_2_O, pH 7.4) was added to 2 mL of EDTA blood. After incubation at room temperature for 20 min, leucocyte pellets were harvested by centrifugation at 3100 rpm for 10 min. After discarding the supernatant, leucocyte pellets were resuspended in 200 μL of Phosphate Buffer Saline (PBS) and stored at −80 °C.

### 2.3. Processing of Milk Samples

Twenty ml of fresh goat milk was poured into a 50 mL Falcon tube and stored overnight at 4 °C. The fat was skimmed from the milk by centrifugation at 3000 rpm for 10 min at 4 °C. If a cell pellet had formed, it was carefully resuspended in the milk with a Pasteur pipette without disturbing the layer of fat that had formed on top. After piercing the bottom of the Falcon tube with a needle, 10 mL of skimmed milk was transferred into a new tube and centrifuged at 4000 rpm for 10 min at 4 °C. Two ml of the supernatant, i.e. lactoserum, was kept for serological testing while the rest was discarded. The remaining cell pellet was resuspended in 2 mL PBS for washing, followed by centrifugation at 4000 rpm for 10 min at 4 °C. The supernatant was removed and the cell pellets were resuspended in 200 μL of PBS and stored at −80 °C.

### 2.4. Serological Analysis by ELISA

The presence of SRLV-specific antibodies in serum and milk samples was tested using Elitest MVV/CAEV^®^ (Hyphen) and IDscreen^®^ MVV/CAEV indirect (IDvet) ELISA kits following manufacturer’s protocols. Each kit uses the same protocol for lactoserum and serum testing, but different predilutions are used of each matrix. In the Elitest^®^, serum and milk samples are prediluted 100× and 10× before testing, respectively. Twenty µL of the prediluted serum/milk are then added to 80 µL of dilution buffer (previously loaded in the testing well). Each testing well finally contains only 0.2 µL of serum or 2 µL of lactoserum in a total of 100 µL. In the IDscreen^®^ ELISA, this is 10 µL of serum in a total of 200 µL, or 50 µL of lactoserum in a total of 100 µL.

Results were only accepted if the internal kit controls fulfilled the prescribed conditions. Samples were considered positive when the optical densities (ODs) in Elitest^®^ or the S/P% values in IDscreen^®^ were equal or above the calculated or prescribed cut-off value, respectively. The formula to calculate the S/P% is provided by the manufacturers, and is the following:

S/P% = (OD_sample_ − OD_NC_ /OD_PC_ − OD_NC_) × 100

(OD: Optical Density, OD_NC_: mean value of the Negative Control OD, OD_PC_: mean value of the Positive Control). Samples classified as doubtful in the IDscreen^®^ MVV/CAEV indirect (IDvet) ELISA were considered as negative for downstream analysis.

### 2.5. Evaluation of the Analytical Sensitivity of ELISA Tests

In order to compare the analytical sensitivity of both ELISA tests, three independent, two-fold dilution series (ranging from 1:4 to 1:1024) were made of a serum sample of an SRLV-positive goat in serum of an SRLV-negative certified sheep and tested in both ELISA kits. The mean and standard deviation of the three repeats for each serum dilution are shown.

### 2.6. DNA Extraction and qPCR Analysis

DNA from leucocytes pellets was extracted using the QIAamp DNA Mini Kit (Qiagen, Hilden, Germany) following manufacturers’ protocol for DNA purification from blood. Briefly, 20 μL of proteinase K and 200 μL of AL were added to leucocyte pellets resuspended in 200 µL. After incubation at 56 °C for 20 min, 200 μL of ethanol (100%) was added to the reaction mixture. Samples were applied to the QIAamp spin column and centrifuged at 8000 rpm for 1 min. The QIAamp spin columns were washed first with 500 μL of buffer AW1, followed by washing with 500 μL of buffer AW2. DNA was eluted in 100 μL of elution buffer and stored at −80 °C until used.

DNA from milk cell pellets was extracted using the QIAamp DNA Mini Kit following manufacturers’ protocol for DNA purification from tissue. This protocol differs from the protocol for DNA purification from blood in the cell lysis step. In brief, 180 μL of ATL and 20 μL of proteinase K were added to 100 μL of milk cell pellets. Samples were first incubated at 56° C for 30 min, and after adding 200 μL of AL, they were incubated at 70 °C for 10 min. Then 200 µL of ethanol was added, and the protocol described above was followed. DNA was eluted in 100 μL of elution buffer and stored at −80 °C. Five μL of eluted DNA was used as starting material in our previously described in house qPCRs [15,33] to assess the presence of SRLV proviral DNA in extracts from leucocyte and milk cell pellets. All samples were tested for the presence of genotype A and/or genotype B strains, and for the presence of β-actin as an extraction control. In each run, also negative extraction and negative and positive amplification controls were included. All qPCRs were carried out on a LightCycler 480 Real-Time PCR system (Roche, Basel, Switzerland) using the FastStart TaqMan Probe Master Mix (Roche, Basel, Switzerland) and the following amplification program: 10 min at 95 °C, followed by 45 cycles of 15 s at 95 °C and 45 s at 60 °C. Samples that had a Ct value > 40, but presented a characteristic amplification curve were considered as positive.

### 2.7. Statistical Analysis

A linear regression analysis was performed to analyze whether the Ct values of the positive leucocyte pellets could be used to predict the Ct values in cell pellets from milk. Data were analyzed using Data analysis tools provided by Excel. P values < 0.05 were considered to be significant.

## 3. Results

### 3.1. SRLV-Specific Antibody Detection in Serum and Milk via ELISA

Serum and lactoserum of 321 goats were first tested in the Elitest^®^ MVV/CAEV ELISA. Table 1 shows that 305 out of 321 samples obtained the same SRLV infection status in serum and in milk. Twelve out of 180 positive samples in serum were found to be negative in milk, leading to a relative sensitivity of 93% in milk versus serum, while four samples tested negative in serum but positive in milk, resulting in a relative specificity of 97%. The 12 presumed false negative milk samples originated from only two out of eight tested farms (#1 and #8), while three out of four presumed false positive milk samples came from farm #2 (see Table S1).

**Figure 1:**
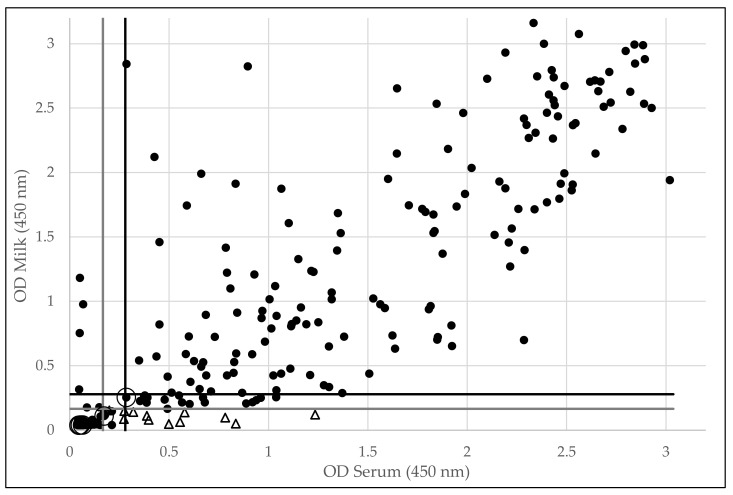
Overview of optical density (OD) values obtained in serum and corresponding milk samples for 321 goats in the Elitest^®^ ELISA. The 12 samples that were positive in serum and negative in milk are indicated by a triangle. Dots surrounded by an open circle indicate samples that got a divergent infection status in the IDscreen^®^ ELISA (see Figure 2). The cut-off OD value of different plates ranged between 0.166 and 0.279, and are indicated by the solid lines.

Figure 1 shows that besides the 12 samples that were positive in serum but negative in milk, additional animals seem to be clearly positive in serum while the OD values in milk remain close to the cut-off value, suggesting that more antibodies are present in serum than in milk.

Somewhat different results were obtained when the same samples were subsequently tested in the IDscreen® MVV/CAEV indirect ELISA (Table 1). A similar number of animals obtained the same SRLV infection status in both matrices, but considerably more serum (201 vs. 180) and milk (194 vs. 172) samples were found to contain SRLV-specific antibodies in the IDscreen® ELISA compared to the Elitest®.

**Figure 2:**
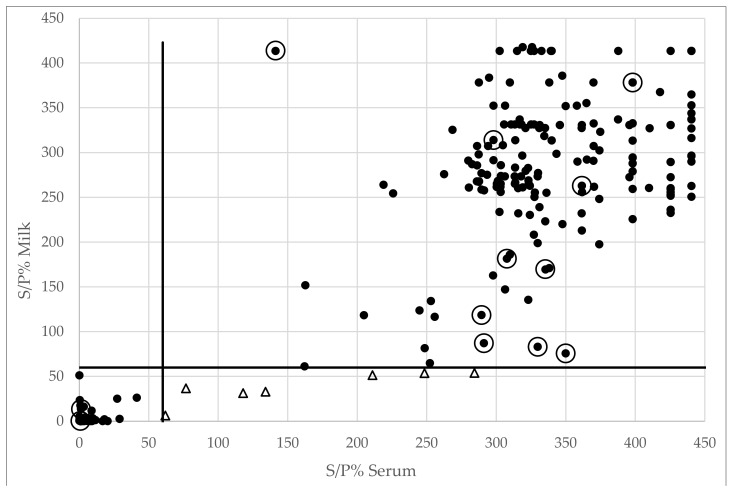
Overview of S/P values obtained in serum and corresponding milk samples in the IDscreen^®^ ELISA. The seven samples that were positive in serum and negative in milk are indicated by a triangle. Dots surrounded by an open circle indicate samples that got a divergent infection status in the Elitest^®^ ELISA (see Figure 1). The cut-off S/P value of 60% is indicated by the full line.

Only 7 out of 201 animals positive in serum were found negative in milk, while all animals positive in milk were also positive in serum, leading to a relative sensitivity and specificity of 97% and 100%, respectively. Again, most of the presumed false negative samples in milk originated from the same two farms (#1 and #8, see Table S1). Also, here some additional animals (to the seven with divergent results) seem to have high S/P values in serum, but S/P values just above the cut-off in milk (Figure 2), further suggesting that sometimes more antibodies are present in serum than in milk.

Interestingly, the four lactoserum positive but serum negative animals in the Elitest^®^ obtained a different infection status in the IDscreen^®^ ELISA. Three of them were found negative in both matrices, while the other was negative in milk and positive in serum. This suggests that these samples represented false positive results in milk in the Elitest^®^. 

Overall, both the IDscreen^®^ (Pearson correlation coefficient: r = 0.92) and the Elitest^®^ (Pearson correlation coefficient: r = 0.89) show a good correlation between the S/P and OD values, respectively, for both serum and lactoserum, and this in negative and positive animals (Figure 1 and Figure 2). Nevertheless, a clearer separation between the population of negative and positive animals can be observed in the IDscreen^®^ ELISA.

The results described above show that considerably more serum and milk samples were found positive in the IDscreen^®^ ELISA than in the Elitest^®^. Supplementary Figure S1 shows that a considerable number of animals show high positive S/P values in the IDscreen^®^ ELISA while remaining negative in the Elitest^®^. To evaluate this in more detail, we tested the analytical sensitivity of both kits using dilution series of an SRLV positive serum sample from a goat in both tests. 

**Figure 3:**
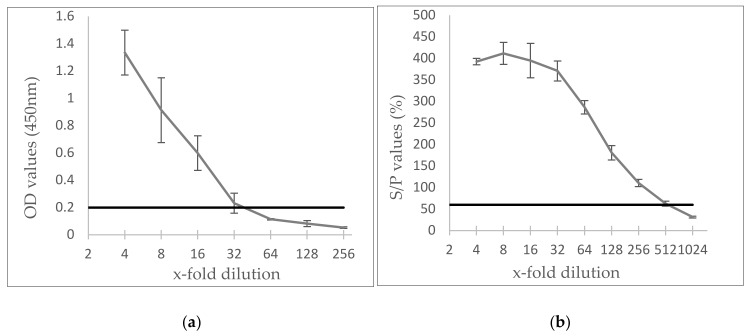
Analytical sensitivity of Elitest^®^ (**a**) and IDscreen^®^ (**b**) ELISA. Three independent 2-fold dilution series of a positive goat sample were prepared in a negative sheep serum and tested in both kits. The cut-off value was 0.199 in Elitest^®^ and 60% in IDscreen^®^.

Figure 3 shows that the Elitest^®^ detected SRLV-specific antibodies until a dilution of 1/32 (2 out of 3 positive) while the IDScreen^®^ ELISA was capable to detect SRLV-specific antibodies until a 1/512 (2 out of 3 positive) dilution. This indicates that the IDscreen^®^ ELISA has a significantly higher analytical sensitivity than the Elitest^®^.

### 3.2. Molecular SRLV Detection in PBMCs and Milk Cell Pellets by qPCR

In order to evaluate the suitability of milk for qPCR-based diagnosis, we compared SRLV detection by qPCR in leucocyte pellets and cell pellets isolated from milk (Table 2). The results show that 272 out of 321 animals obtained the same SRLV infection status in leucocyte and milk cell pellets. This corresponds to 84% concordance between both matrices. Out of 165 peripheral blood mononuclear cell (PBMC)-positive animals, 31 remained negative in cell pellets isolated from milk, leading to a relative sensitivity of 81%. Again, most of these animals originated from farms #1 and #8 (see Table S2). On the other hand, 18 animals that tested negative in PBMCs were however SRLV positive in milk cell pellets, resulting in a relative specificity of 88%. All goats were found to be infected with genotype B strains, while none of the animals was positive in the genotype A qPCR.

**Figure 4:**
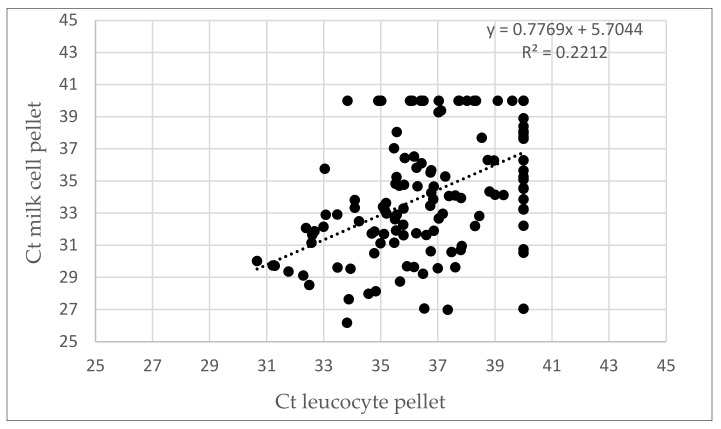
Linear regression analysis of Ct values found in PBMCs and milk cell pellets of animals that were qPCR positive in both matrices.

A significant relationship (slope = 0.7769; P = 9.78 × 10^−09^) between the Ct values of the 134 samples positive in both matrices was found in a linear regression analysis (Figure 4), while the Pearson correlation coefficient r = 0.47 (r^2^ = 0.22) indicates a moderate, positive relationship. This indicates that the Ct values in PBMCs could be used to predict the Ct values in cell pellets from milk. Using the diagnostic methods that we applied, the obtained Ct values in leucocyte pellets were on average 2.47 Cts higher than in milk cell pellets, showing that milk cell pellets contained on average five times more proviral DNA than leucocyte pellets.

## 4. Discussion

In the absence of vaccines to limit economic losses due to SRLV infections in the small ruminant industry, control programs are of crucial importance for the detection and the elimination of SRLV-infected animals. These programs depend on good diagnostic assays that mostly use blood samples [2,13]. When governments implement voluntary control programs, as is the case in Belgium, the participation among sheep and goat owners is often limited due to high costs for blood sample collection and testing [32]. Therefore, we evaluated whether milk, which is more easily and cheaply collected, could be used as a valid matrix for serological and virological SRLV detection.

Antibodies are known to be present in milk. These are mainly derived from serum and are transported across the mammary epithelial cells to milk by transcytosis, but can to a lower extent also be locally produced in the mammary gland [22,23,28]. When comparing SRLV antibody detection in milk to serum using the IDscreen^®^ ELISA kit, a high concordance between both matrices was found with a relative sensitivity and specificity of 97% and 100%, respectively. The divergent results relate to only seven animals that were found positive in serum and negative in milk. This probably reflects the higher concentration of antibodies in serum compared to milk [28,34,35,36,37]. It is likely that these animals were at the late stage of lactation when milk and blood were sampled, since it is known that the amount of antibodies and B lymphocytes in milk decreases as lactation progresses [36,37,38,39]. This seems plausible, since prolonged milking, i.e., continued milking of goats for several years as long as sufficient milk is produced without renewed mating, is regularly practiced in the dairy goat sector in Belgium and the Netherlands.

Our results are thus in line with other studies that have evaluated the usefulness of milk testing for SRLV detection [19,21,29,30] and indicate that milk could be a suitable matrix for SRLV antibody detection. Serum however remains the matrix of choice when it is the purpose to identify each SRLV-positive animal as soon as possible, as is the case in control programs. Although we only analyzed samples from goats, we estimate based on previous studies [19,21,29] that similar results are to be expected also in sheep.

Somewhat less favorable results were obtained when the Elitest^®^ kit was used for antibody detection. Considerably less animals were identified as SRLV positive, both in serum and milk, when compared to the IDscreen^®^ ELISA results. This is most probably due to the better analytical sensitivity that we observed for the IDscreen^®^ compared to the Elitest^®^ ELISA. This could either be due to differences in antigen used in both tests [15] and/or to the 25- and 50-fold higher milk or serum volumes, respectively, that are used per testing well of the IDscreen^®^ compared to the Elitest^®^ ELISA. 

Interestingly, this difference in sensitivity was not observed in our previous study, where both tests were compared using randomly collected sheep and goat serum samples from Belgium [15]. Probably, the recent change in the protocol of the IDscreen^®^ ELISA between both studies has increased the sensitivity of the test.

In addition, more divergent results between SRLV-specific antibody detection in milk and serum were found in the Elitest^®^. Twelve animals were found positive in serum and negative in milk. As indicated above for the IDscreen^®^ ELISA, the most likely explanation is that the number of antibodies in these 12 milk samples is too low to be detected, potentially due to sample collection late during lactation when the amount of antibodies and B lymphocytes has already decreased [28,34,35,36,37]. Interestingly, four other animals tested positive in milk and negative in serum. Based on the results of the IDscreen^®^ ELISA, we consider it most likely that these milk results are false positive, something that has already been described to occur when colostrum, mastitic milk, or milk with excess of fat is tested in ELISA [35,40].

Despite the high relative sensitivity (97%) and relative specificity (100%) obtained when comparing SRLV detection in milk to serum using the IDscreen^®^ ELISA, it is important to keep in mind that there is no gold standard diagnostic method for SRLV [3,18], and that 100% relative specificity does not mean that no false positive results can occur. Serological tools may suffer imperfect sensitivity that may be related to the heterogeneity of circulating strains, delayed seroconversion and fluctuating antibody responses [2,6,16,41,42]. Besides issues with imperfect sensitivity, also false positive reactions are sometimes observed which mostly cannot be explained [43]. We can thus not exclude that the IDscreen^®^ ELISA may not detect SRLV infection in animals infected with phylogenetically-divergent strains or may produce false positive results.

We also addressed the suitability of milk as a matrix for SRLV detection by qPCR. Monocytes carrying provirus infiltrate from blood into the mammary gland where they differentiate into macrophages, leading to viral replication. Our evaluation of SRLV detection by qPCR in milk compared to blood led to mixed results with a relative sensitivity and specificity of 81% and 88%, respectively. This imperfect concordance is probably related to the moment of lactation when samples were collected. CAEV infection is namely associated with alternating periods of reduced viral expression and periods of reactivation which are often associated with the onset of lactation [44,45]. At early lactation, the proportion of macrophages in milk is high, and CAEV replication in macrophages increases [36,46]. Additionally, colostrum is described to harbor a higher proviral and viral load compared to blood [24]. During late lactation, the percentage of macrophages tends to decrease [38,39].

It is likely that the samples from animals that were positive in blood but negative in milk were collected at late lactation. This is supported by the fact that most of these samples originated from the same two farms in which also multiple antibody positive serum but negative milk samples were collected. Another possible explanation would be that the immune response in the mammary gland of these animals was rather efficient, resulting in a reduced viral load in cell pellets isolated from milk [47]. On the other hand, 18 animals tested negative in blood and positive in cell pellets isolated from milk. Probably these were collected during early lactation when a higher proportion of macrophages is present in milk samples compared to blood monocytes. 

Another explanation could be that these animals suffer from mastitis, since this is known to be related to an accumulation of macrophages [48,49]. It has been suggested that infected macrophages enter milk more easily when the mammary gland is inflamed and has severe lesions [50,51]. This last explanation is however less likely for our study, since no clinical mastitis was observed during sample collection.

Although the relative sensitivity of SRLV detection by qPCR was lower in milk than in PBMCs, as explained above, we found that animals that were positive in both matrices contained 5-fold higher amounts of proviral DNA in milk compared to leucocytes. This difference could simply be related to differences in the applied methods (higher sample volume, differences in extraction protocol) to obtain proviral DNA from milk or blood samples, but it could also be linked to the fact that mammary epithelial cells are highly permissive to CAEV infection, and may end up in colostrum/milk during lactation [4,24,39,49,52,53,54]. Mammary epithelial cells can be infected by macrophages, act as virus reservoirs and sustain viral replication [53,55,56], thereby increasing the proviral load in milk.

On top of the mixed results in SRLV detection by qPCR in milk and serum, it is important to notice that virological SRLV detection showed to be clearly less sensitive than antibody detection by IDscreen^®^ ELISA. These results are in line with the previous study of Michiels et al. [15] and advocate to consider qPCR mainly as a confirmatory test. In general, qPCR sensitivity is negatively affected by the low proviral DNA load and the high viral genetic heterogeneity [3], and the isolation of cell pellets from milk is a time consuming and cumbersome process. Animals that tested positive in milk by ELISA and negative by qPCR may suffer from SRLV infection in other organs than the mammary gland, making that only few infected cells are found in milk, while antibodies diffuse nevertheless into the mammary gland [19,20,57].

## 5. Conclusions

When SRLV antibody detection in milk is compared to serum, the IDscreen^®^ Elisa showed to be highly sensitive and specific. A limited number of serum antibody-positive animals was however found negative in milk, probably related to the stage of lactation at which the samples were collected. This makes it true that milk is an easy to collect and cheap matrix for a preliminary evaluation of the herd’s infection status, which can help the farmer to decide whether obtaining an SRLV-free certificate is conceivable. Serum however remains the sample of choice for control programs where it is important to identify SRLV-positive animals as soon as possible with the highest sensitivity. SRLV detection by qPCR in milk tended to be more variable, and is probably even more strongly influenced by the moment of sample collection compared to antibody detection in milk. It was furthermore clearly less sensitive than antibody detection and therefore, serology remains the preferred first line screening method.

## Figures and Tables

**Table 1:** Relative sensitivity and specificity of small ruminant lentivirus (SRLV)-specific antibody detection in milk versus serum via Elitest^®^ and IDscreen^®^ enzyme-linked immunosorbent assays (ELISAs).

**Elitest^®^**						
		**Infection status in milk**				
		Pos	Neg	Total	Relative sensitivity	Relative specificity
**Infection status in serum**	Pos	168	12	180	**93%**	**97%**
	Neg	4	137	141		
	Total	172	149	**321**		
**IDscreen^®^**						
		**Infection status in milk**				
		Pos	Neg	Total	Relative sensitivity	Relative specificity
**Infection status in serum**	Pos	194	7	201	**97%**	**100%**
	Neg	0	120	120		
	Total	194	127	**321**		

**Table 2:** Comparison of SRLV detection by qPCR in peripheral blood mononuclear cells (PBMCs) and cell pellets isolated from milk

PBMCs	Milk Cell Pellets				
	Pos	Neg	Total	Relative Sensitivity	Relative Specificity
Pos	134	31	165	**81%**	**88%**
Neg	18	138	156		
Total	152	169	**321**		

## Supplementary Materials

The following are available online at https://www.mdpi.com/1999-4915/12/1/3/s1, Table S1: Overview of the comparison of SRLV antibody detection in serum and milk samples per farm via Elitest® and IDscreen® ELISAs., Table S2: Overview of the comparison of SRLV detection in leucocyte pellets and milk cell pellets per farm via qPCR, Figure S1: Overview of OD values obtained in Elitest® compared to S/P% values in IDscreen^®^ kit in serum (**a**) and milk (**b**).
