# Supplementary material for: (Non-)Sense of Milk Testing in Small Ruminant Lentivirus Control Programs in Goats. Comparative Analysis of Antibody Detection and Molecular Diagnosis in Blood and Milk
Source: Viruses. 2019 Dec 18;12(1):3. doi: 10.3390/v12010003 (PMC7019267; doi:10.3390/v12010003)
Supplement: Supplementary file 1 [file viruses-12-00003-s001.pdf]

Table S1. Overview of the comparison of SRLV antibody detection in serum and milk samples per farm via Elitest® and IDscreen® ELISAs.

Elitest®

Farms	Total number of lactating animals per farm	Total number of samples selected per farm	Serum/milk are Pos	Serum/milk are Neg	Serum Pos / milk Neg	Serum Neg / milk Pos
1	420	56	44	7	5	0
2	310	40	5	32	0	3
3	45	9	9	0	0	0
4	400	52	40	12	0	0
5	18	13	1	11	0	1
6	360	47	8	39	0	0
7	200	30	3	27	0	0
8	600	74	58	9	7	0
Total	2 353	321	168	137	12	4

IDscreen®

Farms	Total number of lactating animals per farm	Total number of samples selected per farm	Serum/milk are Pos	Serum/milk are Neg	Serum Pos / milk Neg	Serum Neg / milk Pos
1	420	56	54	0	2	0
2	310	40	6	34	0	0
3	45	9	9	0	0	0
4	400	52	40	12	0	0
5	18	13	1	11	1	0
6	360	47	12	35	0	0
7	200	30	3	26	1	0
8	600	74	69	2	3	0
Total	2 353	321	194	120	7	0

Table S2. Overview of the comparison of SRLV detection in leucocyte pellets and milk cell pellets per farm via qPCR.

Farms	Total number of lactating animals per farm	Total number of samples selected per farm	PBMCs/milk cell pellets are Pos	PBMCs/milk cell pellets are Neg	PBMCs Pos / milk cell pellets Neg	PBMCs Neg / milk cell pellets Pos
1	420	56	39	4	13	0
2	310	40	4	34	1	1
3	45	9	9	0	0	0
4	400	52	30	15	3	4
5	18	13	1	12	0	0
6	360	47	10	32	0	5
7	200	30	3	26	0	1
8	600	74	38	15	14	7
Total	2 353	321	134	138	31	18

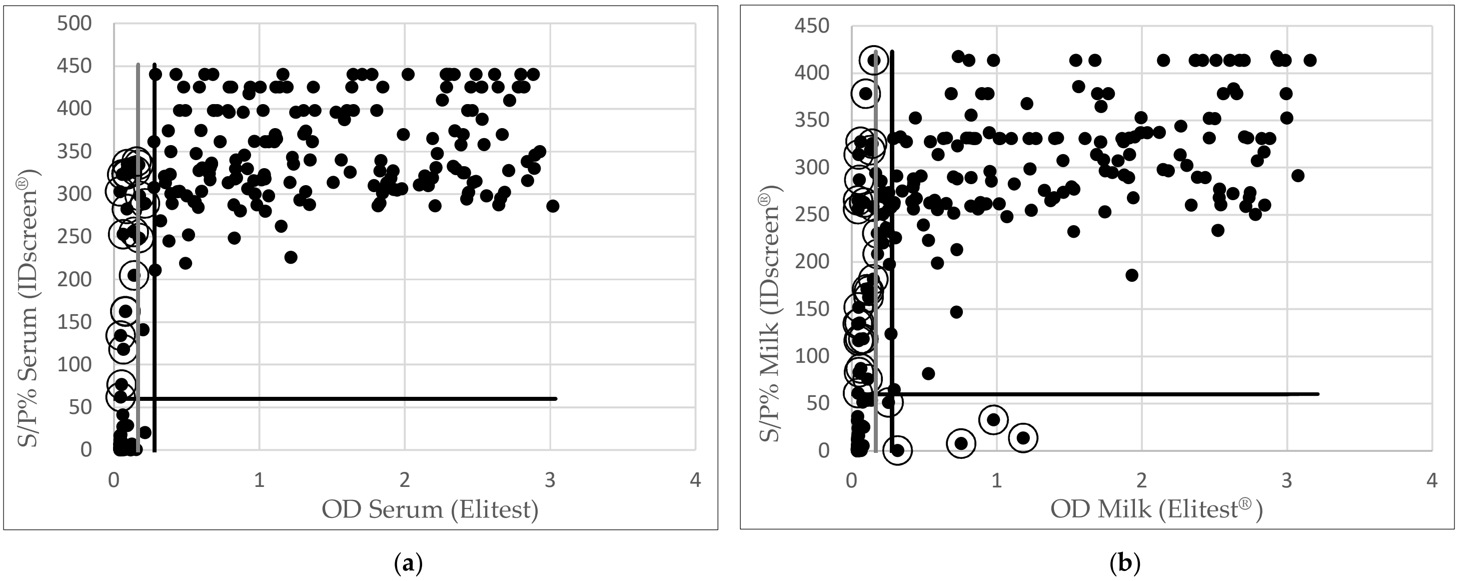

Figure S1. Overview of OD values obtained in Elitest® compared to S/P% values in IDscreen® kit in serum **(a)** and milk **(b)**. The cut-off values of each test are indicated by full lines.
